# BCR::ABL1阳性急性B淋巴细胞白血病分型与预后评估进展

**DOI:** 10.3760/cma.j.cn121090-20240315-00096

**Published:** 2024-07

**Authors:** 宇辰 闫, 成 王, 坚青 糜, 瑾 王

**Affiliations:** 上海交通大学医学院附属瑞金医院血液内科，上海 200025 Department of Hematology, Ruijin Hospital, Shanghai Jiaotong University School of Medicine, Shanghai 200025, China

## Abstract

酪氨酸激酶抑制剂和免疫靶向药物的应用已经明显改变了BCR::ABL1阳性急性B淋巴细胞白血病的治疗策略和临床结果。2022年，世界卫生组织（WHO）和国际共识分型（ICC）相继对该疾病的分型进行了更新。2023年，美国国立综合癌症网络（NCCN）也首次更新了该疾病的预后分层，其治疗后的微小残留病评估和IKZF1^plus^基因分型是最关键的预后因素。以上更新将对临床诊疗产生重大影响，本文聚焦于该疾病分型和预后评估的最新更新内容展开综述。

## Abstract (en)

The application of tyrosine kinase inhibitors and targeted immunotherapy has revolutionized the therapeutic strategies and clinical outcome for BCR::ABL1-positive B-cell acute lymphoblastic leukemia (BCR::ABL1^+^ B-ALL). The classification was updated successively by the World Health Organization and the International Consensus Classification in 2022. The risk stratification of this entity, for the first time, was modified by the National Comprehensive Cancer Network in 2023, both minimal residual disease assessment and IKZF1^plus^ genotyping recognized as critical prognostic factors. These important updates would have significant implications for clinical management. Therefore, this review focused on the latest advances in the classification and prognostic evaluation of BCR::ABL1^+^ B-ALL.

BCR::ABL1阳性急性B淋巴细胞白血病（B-ALL）约占成人ALL的25％[1]，在60岁以上的ALL患者中占比可达40％以上[2]。虽然21世纪以来酪氨酸激酶抑制剂（TKI）的应用极大改善了BCR::ABL1阳性B-ALL患者的预后，且近十年来免疫治疗与TKI的联合应用进一步提高了疗效[3]，但在2022年以前，该亚型一直被各类指南列为细胞遗传学预后不良组[4]。2022年，国际共识分型（International Consensus Classification，ICC）将BCR::ABL1阳性B-ALL分为仅淋巴细胞受累和多系受累两个亚型[5]。2023年，美国国立综合癌症网络（National Comprehensive Cancer Network，NCCN）首次将BCR::ABL1阳性B-ALL分为标危和高危两组。由此可见这些重要更新将对临床诊疗产生重大的影响，但目前尚缺乏对这些更新内容的系统性介绍。因此，本文聚焦于近期成人BCR::ABL1阳性B-ALL的分型和预后评估的进展，结合最新研究成果展开综述。

一、分型

随着对BCR::ABL1阳性B-ALL的发病机制相关的基础研究的深入，WHO第5版血液淋巴肿瘤分型[6]和ICC[5]在2022年对该疾病的分型进行了相应更新。

1. WHO分型：2022年发表的第5版WHO血液淋巴肿瘤分型采纳了国际人类基因组组织基因命名委员会的建议，使用“::”表示基因断裂后融合[7]，同时去除了第4版名称中的染色体核型。原第4版名称为“B lymphoblastic leukemia/lymphoma with t（9;22）（q34;q11.2）;BCR-ABL1”[8]，在第5版更新为“B lymphoblastic leukemia/lymphoma with BCR::ABL1 fusion”[6]。

2. ICC分型：2022年发表的ICC分型根据原位荧光杂交（FISH）实验中代表BCR::ABL1融合基因的荧光点是否仅出现于淋巴细胞中，首次将BCR::ABL1阳性B-ALL分为两类：第一类仅淋巴细胞受累；第二类除淋巴细胞受累之外，髓系细胞也受累[5]。有研究指出多系受累患者可能和慢性髓性白血病急淋变（chronic myeloid leukemia lymphocytic blast crisis，CML-LBC）相关[9]–[10]，据此，NCCN推荐使用FISH鉴别CML-LBC和BCR::ABL1阳性B-ALL[11]。Kamoda等[12]分析了20例BCR::ABL1阳性B-ALL患者的FISH结果，发现仅淋系受累患者白细胞计数在15×10^9^/L左右，抗原跨系表达较少，融合基因p190转录本较多；而多系受累患者白细胞计数一般>100×10^9^/L，常有髓系抗原表达，融合基因p210转录本较常见。Hovorkova等[13]的研究表明，仅淋系累及的患者所具有的BCR::ABL1融合基因出现于白血病原始细胞生成阶段，而多系受累患者的BCR::ABL1融合基因出现于多能造血祖细胞阶段，这解释了为何多系受累患者在其他细胞中也能检测到BCR::ABL1融合基因。

由于临床上FISH的应用限制条件较多，对于不能进行FISH检测的患者，也可以借助聚合酶链反应（PCR）和二代测序（NGS）等分子生物学技术进行分型，并进一步揭示两类患者分子生物学层面的差异。Zuna等[14]在147例BCR::ABL1阳性B-ALL患者第一次诱导治疗结束后，用定量PCR（quantitative PCR, qPCR）检测BCR::ABL1拷贝数和IgH/TCR重排，发现仅淋系受累患者的BCR::ABL1拷贝数和IgH/TCR的变化趋势基本一致；而多系受累患者IgH/TCR明显降低，但BCR::ABL1拷贝数仍维持在较高水平。基于上述结果，该团队使用DNA测序技术分析了884例BCR::ABL1阳性B-ALL患者的BCR基因和ABL1基因断裂点情况，发现仅淋系受累患者的ABL1基因断裂点接近外显子1的3′端，多系受累接近5′端[15]。而Bastian等[16]分析了4个队列共327例BCR::ABL1阳性B-ALL患者，发现仅淋系受累患者最常见的染色体结构变异（SV）是IKZF1缺失与PAX5/CDKN2A缺失，同时伴有淋系抗原（CD20、CD22）高表达；而多系受累患者最常见的SV是HBS1L缺失与7号染色体缺失，伴有髓系抗原（CD13、CD33）高表达。该团队随后在GMALL队列中（98例）比较这两种不同类型患者的预后，发现仅淋系受累患者的3年无病生存（DFS）率为（61±6）％，多系受累患者的3年DFS率为（70±9）％，两者差异无统计学意义（*P*＝0.53）。

二、预后评估

TKI前时代，BCR::ABL1阳性B-ALL被列为高危亚型。其预后极差，3年总生存（OS）率仅能达到13％。第一、二代TKI的应用使其3年OS率达到47％，而第三代TKI和免疫治疗（如贝林妥欧单抗）的联合应用可进一步提高其3年OS率至80％以上[17]。微小残留病（MRD）已经是国际公认的重要预后因子，治疗后MRD转阴的BCR::ABL1阳性B-ALL患者的OS率和DFS率均更高[18]。2023年起NCCN指南开始将BCR::ABL1阳性B-ALL分为高危和标危两组：IKZF1^plus^基因型或伴有慢性髓性白血病（CML）既往史的患者为高危组；非IKZF1^plus^基因型且无CML既往史的患者为标危组[11]。本文主要介绍MRD和IKZF1^plus^在BCR::ABL1阳性ALL预后评估中的作用。

1. MRD：MRD的结果与BCR::ABL1阳性B-ALL患者的预后相关[18]。MRD阳性的患者更容易复发，且预后明显差于MRD持续阴性的患者[19]。定期监测患者的MRD，基于其结果来及时调整治疗的策略，有助于提高疗效和改善患者的长期生存[11]。常见的那些MRD检测方法因具有不同的原理而导致各自检测的深度不同。在不同的检测时间点来监测MRD可具有不同的指导作用及预后意义。

（1）MRD检测方法：目前国内临床上评估BCR::ABL1阳性B-ALL的MRD的主要技术是多参数流式细胞术（multiparametric flow cytometry, MFC）与qPCR。MFC具有快速简便的优势，检测深度能达到10^−4^［20]。治疗前需要先确定白血病相关免疫表型（leukemia-associated immunophenotypes, LAIP），随后在治疗过程中根据LAIP进行MRD监测[21]。相较于MFC，qPCR灵敏度更高[22]，检测深度可达10^−5^［23]。但由于其需要特定的引物，只能检测某些特定的基因异常[24]。此外，BCR::ABL1的激酶结构域（kinase domain, KD）突变会导致患者对TKI耐药[25]，故有必要同时使用qPCR测定BCR::ABL1的拷贝数和KD突变[26]。近年来临床试验中应用较多的NGS主要用于检测IgH/TCR重排，其灵敏度更高[27]–[28]，检测深度可达到10^−6^。NGS可以检测到更多的基因异常[29]，未来可能在临床检测中得到广泛应用。

上述三种MRD检测方法原理各不相同，联合应用MFC、qPCR或NGS可以更加精准地监测MRD，尤其是监测ICC分型中多系受累的患者。Short等[30]同时使用qPCR和NGS两种方法检测了90例BCR::ABL1阳性B-ALL患者诱导治疗后的MRD，发现在16例多系受累患者的骨髓样本中，qPCR测定BCR::ABL1融合基因显示MRD阳性（且使用免疫治疗有效后qPCR复查结果仍为阳性），但NGS测定IgH/TCR重排显示MRD阴性，两种检测方法的结果不一致，间接提示了这些患者属于多系受累亚型。

（2）MRD早期评估的时间点：对于BCR::ABL1阳性B-ALL而言，目前较为公认的早期评估MRD的两个时间点分别是诱导治疗后及第3个月[31]–[33]。Short等[34]在诱导治疗后缓解及第3个月时，将85例初发BCR::ABL1阳性B-ALL患者依据MRD是否转阴分组，比较这两个时间点MRD阴性和MRD阳性患者的预后差异。Cox回归分析显示3个月时达到完全分子学缓解（CMR）是预后的独立保护因素（*HR*＝0.42）。相较于诱导缓解后的时间点［OS：*P*＝0.10，无复发生存率（RFS）：*P*＝0.04］，在3个月时评估MRD的预后意义（OS：*P*＝0.005，RFS：*P*＝0.002）更为显著。Cazzaniga等[35]对151例BCR::ABL1阳性B-ALL患者的分析显示，诱导治疗后10～12周时MRD阴性的患者具有更低的复发率［5年累积复发率（CIR）为14.3％］。这说明在诱导治疗3个月时评估BCR::ABL1阳性B-ALL的MRD更为合适。

（3）异基因造血干细胞移植（allo-HSCT）前后的MRD评估：allo-HSCT前MRD阴性显著降低了allo-HSCT后复发风险。Nishiwaki等[36]对432例BCR::ABL1阳性B-ALL患者的回顾性分析显示，在移植前MRD阳性的155例患者的4年CIR显著高于MRD阴性的277例患者（29％对19％，*P*＝0.006）。而allo-HSCT后仍MRD阳性的患者也更容易复发。Pfeifer等[37]分析了54例BCR::ABL1阳性B-ALL患者移植后的生存情况，发现移植后MRD阳性的30例患者的复发率显著高于移植后MRD阴性的24例患者（29.8％对0，*P*＝0.017）。由此可见在allo-HSCT前后评估MRD均是必要的，有助于评估患者移植后的复发风险和长期生存。

2. IKZF1^plus^基因型：IKZF1^plus^的定义于2018年在两项儿童B-ALL的研究中被首次提出：IKZF1基因缺失的同时伴有CDKN2A、CDKN2B、PAX5和PAR1区中CRLF2、CSF2RA、IL3RA基因中至少一个缺失，且没有ERG缺失[38]–[39]。目前可用于鉴定IKZF1^plus^的方法主要为以下三种：①多重连接探针扩增（multiplex ligation-dependent probe amplification，MLPA）技术[40]是现在的主流方法，利用多对特异长度的探针分别与目标序列配对后连接扩增。通过扩增产物的荧光强度判断靶基因的拷贝数改变[41]。其主要优点是速度快，特异性高，可大批量且半定量测定多种靶基因的缺失。但靶基因序列改变会阻碍探针配对[41]，从而导致假阳性。此外，MLPA的探针决定了其只能检测特定的基因缺失，且在检测IKZF1^plus^时检测深度为0.2[42]。②光学基因组图谱（optical genome mapping，OGM）技术利用识别特定序列的荧光转移酶，在超长分子DNA中加入荧光标记并与模板比对从而分析SV[43]，其读长约为30 kb[44]，分辨率最高为0.5 kb[45]。与MLPA相比，OGM的主要优点可以分辨单条染色体上90％以上的SV[42]，且检测深度可达0.05[45]。其缺点在于检测速度慢，分辨率低于测序[46]。③三代测序技术的原理是当单链DNA分子通过纳米孔，分析各碱基对应的电压变化的差异来测定碱基序列，与文库比对以确定SV。三代测序的检测速度最快可达每秒0.45 kb[47]，最大读长可达2 273 kb[48]，这使其更适合检测0.05～2 kb的大片段缺失[49]。目前三代测序技术可用于检测慢性淋巴细胞白血病的基因异常，其检测基因插入或缺失的成功率为94％[50]；同时，Wang等[51]使用该技术对73例B-ALL患者进行分子分型的准确率可达94.1％，提示三代测序技术具有进行IKZF1^plus^分型的潜在价值。

IKZF1^plus^在儿童患者中的发生率为6％～14.3％[52]–[53]。伴有IKZF1^plus^的BCR::ABL1阳性B-ALL儿童患者的复发风险会大幅增加，尤其是诱导后MRD持续阳性的患者[54]。Stanulla等[38]分析了1 408例儿童B-ALL患者（其中81例为IKZF1^plus^）的EFS和CIR，发现IKZF1^plus^患者的5年EFS率为（53±6）％，CIR为（44±6）％，预后显著差于仅IKZF1缺失患者［EFS率（79±5）％，CIR（11±4）％］和野生型患者［EFS率（87±1）％，CIR（10±1）％］。

在成人患者中IKZF1^plus^的发生率明显高于儿童。Acosta等[55]分析了45例成人B-ALL患者的基因改变情况，发现其中20例（44％）为IKZF1^plus^，在8例BCR::ABL1阳性B-ALL患者中有4例（50％）为IKZF1^plus^，28例MRD阳性患者中有18例（65％）为IKZF1^plus^。Creasey等[56]开展的一项针对成人B-ALL患者的研究中，77例患者中有28例为IKZF1^plus^，其中13例为BCR::ABL1阳性。目前各种临床研究的结果都显示IKZF1^plus^患者的预后明显较差。Fedullo等[57]分析了使用伊马替尼或达沙替尼联合激素诱导治疗的116例初诊成人BCR::ABL1阳性B-ALL患者，发现IKZF1^plus^患者的36个月DFS率为24.9％，显著低于仅IKZF1缺失的患者（43.3％）。Chiaretti等[58]开展的另一项达沙替尼联合激素治疗60例BCR::ABL1阳性B-ALL患者的临床研究中，IKZF1^plus^患者5年OS率为20％，5年DFS率为0，明显不如非IKZF1^plus^的患者（5年OS率69.5％, 5年DFS率60％）。MD Anderson中心倾向性分析对比了在hyper-CVAD方案基础上，联合应用达沙替尼（50例）和普纳替尼（50例）的BCR::ABL1阳性B-ALL患者，发现达沙替尼对IKZF1^plus^患者和非IKZF1^plus^患者效果均较差（5年OS率40％～44％，CIR 60％），而使用普纳替尼时，IKZF1^plus^患者的5年OS率为62％，CIR为52％，明显差于非IKZF1^plus^的患者，这说明普纳替尼对非IKZF1^plus^患者的疗效优于达沙替尼，但其无法解决IKZF1^plus^患者易复发的问题[59]。Foà等[60]的研究表明，在使用达沙替尼和贝林妥欧单抗联合治疗时，11例IKZF1^plus^患者的1年DFS率为64％，1年OS率为82％，1年CIR为27％，治疗反应明显差于野生型患者（1年DFS率100％，1年OS率100％，1年CIR 0）和仅IKZF1缺失患者（1年DFS率92％，1年OS率93％，1年CIR 8％）。其团队随后开展的普纳替尼和贝林妥欧单抗联合治疗的研究中，有一例IKZF1^plus^患者在CR 3个月后早期复发[61]。Onizuka等[62]分析了21例BCR::ABL1阳性B-ALL患者（10例为IKZF1^plus^）在allo-HSCT后的复发情况，发现allo-HSCT后2年内有4例患者复发，均为IKZF1^plus^，提示allo-HSCT仍未能解决IKZF1^plus^组容易复发的问题。由此可见，常用的TKI对IKZF1^plus^治疗效果均欠佳，即使双特异性抗体也未能显著改善IKZF1^plus^患者的生存。

IKZF1^plus^亚型复发率高的原因仍不清楚[57]，Palmi等[63]使用OGM分析了71例仅IKZF1缺失患者和64例IKZF1^plus^患者后发现，IKZF1^plus^亚型复发率高可能是因为ETV6::RUNX1等预后良好的因素只出现在仅IKZF1缺失的患者中。详细情况尚需进一步的深入机制研究阐明。

三、总结与展望

传统的分型与预后评估方法已经逐渐不能满足BCR::ABL1阳性B-ALL的精准治疗需求。ICC将BCR::ABL1阳性B-ALL分为仅淋系累及和多系受累两型，两者的发病机制和预后均有差异。BCR::ABL1阳性B-ALL的预后因子主要是MRD状态和IKZF1^plus^基因型。但目前尚缺乏同时综合两者进行预后分析的研究。综上，随着BCR::ABL1阳性B-ALL相关机制研究的不断深入，测序技术等新方法的普及与成熟，新药时代下BCR::ABL1阳性B-ALL的分型与预后评估将会更加优化，有利于对患者进行个体化的精准治疗，进一步改善患者的长期生存。
